# Palliative care in the community – the role of the resource nurse, a qualitative study

**DOI:** 10.1186/s12904-021-00860-w

**Published:** 2021-10-14

**Authors:** Håkon Johansen, Ann Karin Helgesen

**Affiliations:** Faculty of Health and Welfare Sciences, Østfold University College, 1757 Halden, Norway

**Keywords:** Resource nurse, palliative care, ethics, nursing home

## Abstract

**Background:**

Approaches involving resource nurses have been used in several fields of practice to enhance quality of care. A literature review reveals limited research on the role of the resource nurse in palliative care in the community.

**Aim:**

To explore experiences related to the role of the resource nurse in palliative care in the setting of nursing homes in Norway.

**Design:**

The study has an explorative design with a qualitative approach.

**Methods:**

Two semi structured group interviews were conducted. Five resource nurses participated in the first interview, two resource nurses participated in the last interview. The group interviews were audiotaped, transcribed verbatim and analysed with systematic text condensation.

**Results:**

The resource nurses wish to promote high-quality palliative care. They are skilled palliative nurses working clinically, and they use their experience and knowledge to talk about and demonstrate good practice. By conveying knowledge and being role models, they bolster their colleagues’ confidence and skills in palliative care and contribute to a shared view of quality. They can potentially play an important role in facilitating reflection and collaboration in the palliative care team. However, the resource nurse’s function is affected by interpersonal, managerial and organisational factors.

**Conclusion:**

The resource nurse most important tool in promoting high-quality palliative care may be to support their colleagues being a role model, and sharing knowledge and experience. The resource nurses play an important role in facilitating reflection and collaboration in the palliative care team and may contribute to ethical awareness and proper dialogues about end of life issues.

## Background

It is estimated that some 40 million people worldwide need palliative care each year, but only about 14% of the people who need palliative care actually receive it. The global need for palliative care is growing as a result of the rising burden of noncommunicable diseases and an ageing population [1].

The major barrier to proper care is limited training in palliative care along with a lack of awareness concerning what palliative care is and its importance for patients [1, 2]. Palliative care aims to prevent and relieve suffering by early identification, assessment and treatment of pain and other problems. In addition, it aims to improve quality of life for both patients with life threatening illness and their families [3]. An ethical dimension of palliative care is the nurse’s obligation to promote health and alleviate suffering [4] and to assist people to “live fully throughout their lives until the end” [5].

In Norway, the quality of palliative care is considered to be generally good, but somewhat variable. Both healthcare personnel and patients report a need to enhance health care personnel’s competence [6, 7]. The national strategy for palliative care [8, 9] recommends appointing a resource nurse at each nursing home and at each home health care district. The appointed nurse is supposed to act as a resource for the patient and next of kin. The resource nurse is also supposed to work with system improvements related to routines, procedures and pathways, in addition to advising and guiding colleagues, disseminating new knowledge and initiating reflection [8]. A parallel term for the resource nurse in the Norwegian context is, in the international literature, the link nurse. The link nurse role is established internationally and are used in many clinical settings included nursing homes. The role of the link nurse aims at bridging the gap between the specialist and the generic nurse, and enforcing evidence-based practice. Even though, the link nurse role is not distinctively defined [10, 11].

Nurses in general learn and develop competence during the course of their work [12] and by working together with more experienced colleagues. Structural factors such as staffing and organisation of the services are important as well to build competence [13, 14]. Previous studies describe empowerment of nurses as an aspect of educational efforts in palliative care that may well result in competence development and positively affect the relationship between nurse and patient [15]. Empowered nurses are more eager to involve the patients in daily care and decision-making, and may become more confident in communication with patients [15].

In palliative care, interprofessional collaboration is considered necessary due to the patient’s complex needs. However, interprofessional collaboration is challenging because of issues involving role understanding, communication and sharing of power [16]. Ethical issues often occur in palliative care and may cause difficulties in the relationship between staff groups, eg nurses and physicians. However, there is a lack of organisational support for resolving ethical issues [17].

A literature review reveals limited research on the role of the resource nurse in palliative care in the community context. Research on this topic in Norway is scarce. Knowledge of the role of the resource nurse might shed light on how the resource nurse can contribute to improve practice and the premises of effective role performance. Therefore, the aim of this study was to explore experiences related to the role of the resource nurse in palliative care in the setting of nursing homes in Norway.

## Methods

### Design

The study has an explorative design with a qualitative approach as research about the resource nurses’ role in palliative care in the nursing home context is limited [18]. Group interviews were used to collect data. A group interview can potentially give more rich data and better reveal the variation in data than an individual interview [19]. In total two semi structured group interviews were conducted over a seven-month period in 2017–2018, at two nursing homes in a county in South-East Norway. The sampling of informants was purposeful and consisted of experienced resource nurses. Criteria for participation were to work as assistant nurse or registered nurse and have the role as resource nurse in the nursing home. Every resource nurse at the two nursing homes, 16 in number, were invited to participate in two group interviews. The first interview was carried out before the start of a project aiming to enhance reflective practice (November 2017), and the second interview was carried out after seven months when the project was completed. The project consisted of a one-day workshop during which the resource nurses reflected and jointly drafted a description of the resource nurse’s role. An external expert facilitated during the workshop. Afterwards, a general description of a resource nurse role was anchored in the quality system of the nursing home. It was decided that the resource nurses were to promote reflection among their colleagues on their own ward during the project period, and the project coordinator (the first author) would support the resource nurses with individual conversations.

### Data collection

The first interview was conducted by a consultant from the development centre for nursing homes and home care services in the county. The second interview was conducted by the second author. An interview guide was used by the authors and contained questions about how the resource nurses perceived their role, the challenges they are confronted with, how they motivate and counsel their colleagues, and what they need to do a good job. The moderator in both interviews directed questions to the group and prompted interaction in the group and discussion on the topic among the group members. This seemed to yield rich data with variation in experiences Table 1.

**Table 1:** Interview guide

Can you please tell about how it is to be a resource nurse in palliative care?	
Which challenges do you face?	
Can you tell about a situation where you have motivated and/ or supervised a colleague, please?	
Do you have experienced situations with supervision that have been not so successful? If yes, please tell me about it.	
What do you need to perform well in the role as a resource nurse?	

### Data analysis

The group interviews were audiotaped, transcribed verbatim and analysed with systematic text condensation according to Malterud [20]. This method entails the condensation of meaning units following development of categories. Our text material, coded into categories, provided the basis for preparing analytical texts that in turn resulted in meaningful descriptions of the different categories in the material.

In the first step of the analysis the transcribed text was read several times, and preconceptions were tentatively set aside. Six temporarily themes were noted.

In the second step of the analysis a thorough scrutiny was made for text elements in the transcription which could contribute to answer the research question. The six preliminary themes were refined into four categories, and the meaning units were coded using these four categories. Distinct phenomena were categorized in different codes, and aspects of the same phenomenon were categorized in the same code. Similarities and differences between and within the categories were reflected upon. Meaning units seemingly not fitting into categories, prompted questions and decisions about changing categorization throughout the analysis. In the third step of analysis the text was decontextualized writing new texts with text elements from the same category i.e., text condensation.

In the fourth step of analysis the text was recontextualized into a coherent text that reflected the meanings of the participants through writing an analytical text based on the text condensations. To be sure that the analytical text reflected the meanings of the participants, the original transcription was scrutinized looking for meaning units that could challenge the analytical text. This last step of the analysis resulted in some minor corrections of the analytical text.

The analytical text, along with illustrating quotations, will be presented in the results Table 2.

**Table 2:** Examples of the analytic approach

Excerpt of meaning unit	Codes	Code group *Condensed unit	Description
*A physician examines the medical data and says “now we have to (transfer the patient to the* *hospital)”. If you have weight and experience to say that you disagree* *with that decision, then you will get a discussion where the physician must do a (re-)* *consideration. But if you don’t have the knowledge, you don’t have the experience, you don’t* *have the guts, then maybe there won’t be any (discussion).*	Cooperation with the physician Expressing and tolerating differences of opinion Showing courage	The importance of collaboration in the palliative care team * *The ballast of knowledge, experience and guts gives confidence to speak up against decisions you can’t agree with. Then you can be ready to discuss them with someone with a higher rank than you.*	*The resource nurse shows courage in interprofessional collaboration, applies her competence and presents arguments.*

## Results

The five resource nurses who participated in the first group interview were three registered nurses (RNs) and two assistant nurses. The participants were four women and one man, and their ages ranged from early 20s to early 60s. Their experience from health care varied from a few years to more than 30 years, and they all worked in nursing homes. Those two resource nurses who participated in the last interview had also participated in the first interview. They were one nurse and one assistant nurse, both female and both experienced nurses. Both interviews lasted 1.5 hours.

### Wish for promoting high-quality palliative care

#### Sustaining knowledge and experience

The data showed that the participants perceived that their colleagues, and students in practical placement as well, change their view of palliative care and gain more interest in the field when, as resource nurses, they conveyed knowledge and experience in palliative care. They also experienced that they could ease the anxiety of nurses having less experience in palliative care.

*«An assistant nurse student (…) she was very insecure about caring for dying patients and everything (…) Then there were a lot of questions we talked about. She said it had affected her and that she had become more interested in this subject, and doesn’t think it was that scary any longer*”.^1^

The data showed that the resource nurse is able to support the next of kin and contribute to good memories when they follow their loved ones during the last days of life. However, the resource nurse may face uncertainty in their role. Knowledge of palliative care and good communication abilities were highlighted as prerequisites to being able to handle the different situations properly.

#### Communicating good practice

It was further described that competence development is crucial to achieve high-quality palliative care. New colleagues and students need teaching, and routines must be observed. The participants used their experience and knowledge to talk about and demonstrate good practice.

According to the participants, the members of the palliative care team (in this context: physician, nurses and assistant nurses) need to have a uniform, shared view of quality to be able to share the same professional goals and a common language of palliative care.

*«To work with competence is crucial (….) to have a shared view of quality about what is to be done (…) and that we are able to speak the same language”.* ^2^

### Challenges with reflection in the palliative care team

#### The virtue of reflection

The data highlighted the inherent need for ethical reflection in palliative care. The personnel are affected by the work and need time to reflect, and reflection makes colleagues more conscious of what they are doing. If the palliative care team does not reflect, the quality of the work is at risk. Reflection in the palliative care team facilitates reaching a common view of central issues in palliative care, eg nutrition and hydration when the patient is close to death.

*“I think it is great working together with others who hold the same basic view. We discuss, we might disagree; that is the way it should be, but we basically agree on how we want it to be”.* ^3^

The data showed that when receiving a discharged patient from the hospital, brief information like the diagnosis and need of care provides how nurses and physicians understand the patient. In such situations the palliative care team needs to cooperate to reach a broader understanding of the patient and their situation.

*“It’s very exciting to hear why you do things that way or what you were thinking to reach that conclusion”.* ^4^

### Spontaneous reflection and planned reflection

The data showed that the resource nurses were enthusiastic after the workshop facilitated by the external expert, and that they were dedicated to performing reflection regularly. Even though, the data showed it turned out to be difficult performing reflection regularly as the colleagues of the resource nurse yielded resistance when it came to systematic reflection. The planned and systematic reflection may invoke fear in the participants, and the structure itself, eg turn taking, can impede the reflection. Spontaneous reflection about specific, experienced situations facilitates better conversations than general discussions about professional or ethical issues. Regardless of the benefits of spontaneous reflection, the data showed that the resource nurses experienced the power in systematic reflection when they were supervised by the external expert at the workshop. The data described that the structure in the systematic reflection, eg taking turns in a roundtable discussion, gives everyone a chance to speak – both those who speak easily in groups and the quiet participant who has to be gently prodded to make his or her opinion known. The members of the palliative care team perceive situations differently, and with open-ended questions, it is possible to talk through difficult situations as well as good situations. The participants stated that whereas the resource nurse normally wants regular reflection sessions, the members of the palliative care team more often reflect spontaneously in situations, or after report, rather than systematically according to a plan. Nevertheless, the reflection related to “here and now situations” has the potential to be both systematic and deep. Reflection in general provides an opportunity to enhance one another’s skills and to become stronger as a team.

The data also showed that it frequently happens, when reflection is scheduled, that something comes up and the reflection is postponed. It was mentioned that the palliative care team ought to maintain a positive attitude towards reflection. If a team member has a negative attitude towards it, the resource nurse may become disheartened.

### The importance of collaboration in the palliative care team

#### Collaboration with the physician

The data described how collaboration in the palliative care team can potentially affect quality of care. Over time, resource nurses and the nursing home physicians share common experiences and reach a shared consensus, eg regarding alleviation of pain. When the nursing home physician has knowledge about the patients and the palliative care team share a common view, collaboration is easy. In situations where the nursing home physician is not available and the resource nurse has to consult a physician from the casualty ward, who is often pressed for time, the casualty physician usually does not know the patient or the situation. In cases like these, collaboration is far more difficult. The resource nurses try to avoid calling the casualty physician and instead plan ahead together with the nursing home physician. When a patient situation changes, they know what to do, eg to avoid transfer to hospital when relief is possible in the nursing home, the informants said.

They also said that resource nurses and physicians occasionally have differences in opinion. The physician may want to continue life-prolonging treatment, but the resource nurse recalls, after several conversations with the patient, that he has expressed that he does not want to live any longer. According to the informants, some physicians leave little room for dialogue, while others are more open minded. The resource nurse shows courage in interprofessional collaboration, applies her competence and presents arguments in a respectful way. The participants defend the nurse’s perspective and what they perceive as the best interest of the patient, but in the end the resource nurse respects the physician’s decision.

*“Sometimes there is a little overtreatment (…) if you have enough experience to say that you disagree, you get a discussion where a physician must reconsider his or her decision (…) but if you don’t have the knowledge, you don’t have the experience or you don’t have the courage, then it won’t be any discussion”.* ^5^

The data from the interviews revealed that the nursing home physician has a limited amount of time on the ward and that the resource nurses take care of conversations with the patients and family relatives that they consider is the physician’s responsibility.

*“I wish the nursing home physician would stay here longer, that we had more access to him, that he had more time to visit all the patients, but this is how things are and we have to make the best of it”.*^6^

They perceived that the physician acknowledges the resource nurse’s competence and trusts the resource nurse, but the resource nurses often want the physician to be more present in conversations about medical issues. The data indicated that their competence can at times be stretched to the limit when it comes to administration of medicines and information to next of kin.

#### Collaboration with colleagues

The data showed that nurses occasionally have different opinions about assessment of the patient and his or her condition. In situations involving ethical dilemmas, nurses may have differences of opinion as to how they interpret the situation and what action to take. The resource nurse may contribute by listening to their colleagues and reach a consensus through discussions, according to the informants.

The data also revealed that the resource nurse has to manage inappropriate attitudes. Professional routines and procedures go out-of-date, but some nurses keep doing the work as they always have done. Especially the personnel who have the longest service tenure do not want to listen to new thinking. The resource nurses perceived, however, that they could positively affect their colleague’s attitudes if they adopted a respectful manner.

### Factors affecting the function of the resource nurse

The data showed that the ward nurse affects the function of the resource nurse, as they have to prioritise competence maintenance in the palliative care team. In addition, it is important that the ward nurse appreciates the work of the resource nurse and allocates time so that he or she is able to perform well. It is stressed that mutual trust between ward nurse and resource nurse is a prerequisite.

*“(The ward nurse) (….) must be able to see the value of it (the work of the resource nurse), the common goal that this work enables good nursing performance”.* ^7^

The data showed that days when there is a shortage of staff make it difficult for the resource nurse to deal with problems, compared to days with proper staffing.

The data showed that the network of resource nurses contributes to building competence and enhances team spirit.

*“I think we need unity; I think we need these network meetings. We need to (….) become aware about what we are supposed to supervise others in doing or initiate in others”.* ^8^

## Discussion

The aim of this study was to explore experiences related to the role of the resource nurse in palliative care in the setting of nursing homes in Norway. The results showed that the resource nurses wish to promote high-quality palliative care and that they potentially can play an important role in facilitating reflection and collaboration in the palliative care team. However, the resource nurse’s function is, affected by interpersonal, managerial and organisational factors.

The results showed that the resource wish to affect their colleagues by being role models and conveying knowledge and experience, and besides, they wish to contribute towards high-quality services through their clinical work and collaboration in the palliative care team.

Learning in the context of professional work entails much more than formal and planned learning. A large part of the essential knowledge needed to perform professionally is tacit and inherent in practice. Much of this kind of knowledge is learned non-formally [13] and the role model plays an important role. Witkamp et al. [21] claim that the goal of training palliative care nurse champions is for them to become ambassadors and role models for high-quality palliative care.

The results indicate a collective practice development in the palliative care team by achieving a shared view of quality and goals and by sharing a common language of palliative care. This is in line with Eraut [13] who described learning in professional work as a collective process. Practice development is a continuous process for better quality care, and it is characterised by several factors, including work-based learning where the practitioners learn in and from practice [22]. Especially palliative care training for unexperienced assistants, can contribute to improve the collective knowledge and attitudes regarding palliative care [23]. The International Council of Nurses’ (ICN ‘s) Code of Ethics states that nurses have a responsibility for nursing practice and therefore should maintain their competence by continually learning. In addition, nurses ought to take the bulk of responsibility for “determining and implementing acceptable standards of clinical nursing practice” [4].

The results pointed to the ethical dimension of palliative care and indicated that reflection affects the strengths of the palliative care team and quality of care. Söderhamn et al. [24] reported that quality of work is improved by reflection. Being critical towards one’s own practice and performing ethical reflections helps to strengthen ethical awareness and provide better care, specifically more person-centred care [24]. It seems reasonable to suggest that ethical awareness is a prerequisite for ethical behaviour. The ICN’s Code of Ethics states that nurses ought to strive for a practice culture that promotes ethical behaviour [4].

The results indicated that the resource nurses experienced the power in systematic reflection at the workshop facilitated by the external expert, and that they were dedicated to performing reflection regularly. Contrary to this experience, the results indicated that the colleagues in palliative care prefer spontaneous reflection during the workday, and some find the planned reflection bothersome even though the systematic and planned reflection more effectively facilitates everyone having their say. Söderhamn et al. [24] reported that the staff finds reflection during the workday to be important, as it permits reflections on ethical dilemmas when they occur. The results showed that the here-and-now reflections have the potential to be both systematic and deep. Sandman et al. [17] reported that colleagues tend to cluster with peers who think in a similar way, and in this perspective, it is reasonable to suggest that prevailing opinions are not challenged.

The results showed that by talking about and demonstrating good practice, the resource nurse is able to help colleagues with little experience from palliative care to gain more confidence and interest. In addition, the results showed that the resource nurse supports the colleagues and helps them to do their best. Söderhamn et al. [24] reported that ethical reflection gave the staff an incentive to learn more in order to give the best possible care. Development of practical work is also characterised by pursuing the practitioner’s best performance – to “allow opportunities of human flourishing to emerge” [22]. When the nurse with little experience becomes more interested and confident in palliative care, it may seem reasonable to suggest that she is on her way to flourishing and better performance.

The results indicated that collaboration in the palliative care team affects quality of care. McDonald and McCalling [16] stated that interprofessional collaboration is important due to the patient’s often complex situation. Champion-Smith et al. [25] reported important benefits of interprofessional collaboration in palliative care. For instance, the physician, through dialogue with nurses, could be able to think beyond the medical model [25]. Interestingly, the results indicate that the physician’s knowledge of the actual situation and patient is a prerequisite for collaboration and high-quality care.

The results indicate that the resource nurses and physician occasionally have differences of opinion, but with knowledge, experience, and courage, the nurse is able to become an advocate for the patient. Whatever the case may be, the nurse respects the decision of the physician after having a dialogue. Collaboration between nurses and physician is a relationship presenting ethical challenges, eg communication and decisions about life prolonging treatment [17]. The ICN’s Code of Ethics states that nurses ought to work for a collaborative and respectful relationship with co-workers [4]. LeBaron et al. [26] reported that the palliative care resource nurse (PCRN) was uncertain and hesitated to approach and talk with the physician about eg goals of care, but a PCRN training made them more confident in advocating for the patient.

It is disturbing that the results showed resource nurses taking on responsibility that exceeds their competence due to physicians’ time constraints. As to conversations that are normally led by the physician, this may be ethically problematic because patients and next of kin need sufficient and accurate information to make choices [4], eg about goal of care. Insufficient information may impair patient autonomy indirectly, because it makes it difficult for next of kin to participate in decisions pertaining to the choices of care that benefit the patient and the choices of care that cause no harm [27]. The ICN’s Code of Ethics [4] states that nurses ought to “use judgement regarding individual competence when accepting responsibility”. Both nursing home physicians and nurses play a key role in conversations with seriously ill patients and their close family relatives [28, 29]. Maybe the answer to this ethical challenge is not simply more presence on the part of the physician, but more time for both nurse and physician in collaboration with palliative care eg in conversation with next of kin.

The results showed that resource nurses sometimes found it challenging to influence colleagues who were not interested in new thinking, but that he or she was able to positively affect their colleagues. This is in line with Heals [11], who reported that some personnel in nursing homes are reluctant to change their practice. Established routines and ways of thinking in the organisation can be a hindrance to new thinking and new practice and may perpetuate outdated practices [30].

The results showed that the resource nurses wish to promote high-quality palliative care. Witkamp et al. [31], in a controlled before-and-after study, reported that implementation of a model with palliative care champions in a hospital resulted in no difference in quality of care during the last days of life. However, the resource nurse may contribute to high-quality palliative care through directly influencing the care, and indirectly by influencing colleagues [8, 9, 11]. The key to better quality may be an increased effort to build competence within the service, eg with a champion nurse team. The care nurse champions are in a strong position to disseminate knowledge and improve practice because they have insight into the ward culture and have many opportunities for practice improvement initiatives [31]. Maybe it is reasonable to suggest that the proactive work of the resource nurses in affecting their colleagues is their most important tool in promoting high-quality palliative care?

The results of this study reveal that both available time and organisation of the palliative care team constrains the function of the resource nurse. Kamal et al. [32] , in the context of hospitals, stated that implementing a care champion network to improve quality requires system changes as well, eg allocating necessary time to talk with the serious ill patients about goals of care. The results showed that support from the ward nurse, and mutual trust between ward nurse and resource nurse is important. Frogatt et al. [33] reported the importance of manager support if one is to succeed with a link nurse system. Witkamp et al. [31] stated that the ward management ought to “acknowledge, facilitate and support” the care nurse champion.

The results of this study also described that the resource nurse her/himself may experience uncertainty, and that skills and knowledge of palliative care are prerequisites for confidence. The results described that the network of the resource nurses builds competence and enhances the team spirit of the resource nurses. Kamal et al. [32] described the care champion network as the place where the generalist level in healthcare meets the specialist level, and the care champions become the link between the specialist level and the generalist level with the purpose of managing quality improvement efforts. When the resource nurses are organised in a team and participate in training, they are empowered by specialist knowledge and become more confident in caring for the dying patient. Furthermore, they share experiences and frustrations [26].

## Study strengths and limitations

The study has a limited number of participants. In addition, experiences of the resource nurse’s role in palliative care in nursing homes is explored only from the perspective of the resource nurse’s themselves (self-report) which could represent a weakness in the study. Five persons participated in the first interview, three of them dropped out at the time of the second interview. The researchers have no information why participants dropped out. Information about reasons for drop-out could have been valuable for the study.

The participants were resource nurses and working in a nursing home, and most of them knew each other. Use of homogenous groups in group interviewing can be purposeful [19, 34].

## Conclusion

The results showed that the resource nurses wish to promote high-quality palliative care through sustaining knowledge and experience and communicating good practice. The results also showed that resource nurses potentially can play an important role in facilitating reflection and collaboration in the palliative care team. Through reflection, the resource nurse may contribute to ethical awareness. With knowledge, experience and courage they are capable of challenging a professional point of view eg about the goal of care. The resource nurse function, however, is affected by and subject to interpersonal, managerial and organisational factors.

## Data Availability

The datasets used and/or analysed during the current study are available from the corresponding author on reasonable request.

## Abbreviations

ICN: The International Council of Nurses
NSD: Norwegian Centre for Research Data
PCRN: The palliative care resource nurse
RN: Registered nurse
